# Collapse Ratio and Operative Duration as Key Predictors of Postoperative Complications Following Cranioplasty

**DOI:** 10.7759/cureus.96932

**Published:** 2025-11-15

**Authors:** Rudra N Shah, Alok Jha, Yam B Roka, Ashish J Thapa

**Affiliations:** 1 Neurosurgery, University Hospitals Coventry and Warwickshire NHS Trust, Coventry, GBR; 2 Neurosurgery, Neuro Cardio and Multispeciality Hospital, Biratnagar, NPL; 3 Neurosurgery, Gandaki Medical College, Pokhara, NPL

**Keywords:** collapse ratio, cranioplasty, decompressive craniectomy (dc), postoperative complication, seizure

## Abstract

Background

Cranioplasty (CP) after decompressive craniectomy (DC) restores cranial integrity, cerebral blood flow, and CSF dynamics, often improving neurological recovery. However, postoperative complications remain a concern, with debate traditionally centered on CP timing. Emerging evidence suggests that radiological parameters such as collapse ratio, the degree of parenchymal depression at the craniectomy site, and operative duration, a marker of intraoperative complexity, may better predict outcomes. This study explored these factors in relation to post-CP complications.

Methodology

We retrospectively reviewed 100 patients (≥16 years) who underwent CP following DC at a tertiary neurosurgical center in Nepal (2018-2024). Patients with prior CP, inadequate imaging, or intracranial tumors were excluded. The collapse ratio was measured on preoperative CT using 3D-Slicer software, and the operative duration and the DC-CP interval were recorded. Postoperative outcomes within six months, including seizures, infection, hemorrhage, hydrocephalus, and revision surgery, were documented. Data were analyzed using t-tests, chi-square/Fisher’s exact tests, logistic regression, and receiver operating characteristic (ROC) curves.

Results

Complications occurred in seven (7%) patients, all manifesting as postoperative epilepsy. The complication group had a higher mean collapse ratio (0.78 ± 0.16 vs. 0.36 ± 0.14, p < 0.001) and a longer operative duration (139.7 ± 19.2 vs. 115.1 ± 14.5 minutes, p < 0.001). Logistic regression identified collapse ratio (OR 1.87, p = 0.001) and operative duration (OR 1.68, p = 0.001) as independent predictors, whereas timing was not significant. ROC analysis showed excellent predictive ability for the collapse ratio (area under the curve (AUC) = 0.812) and moderate predictive ability for operative duration (AUC = 0.702).

Conclusions

A higher collapse ratio and longer operative duration independently predict early post-CP complications, whereas fixed timing thresholds provide limited prognostic value. Incorporating these measures into preoperative assessment may enhance surgical planning and risk stratification, particularly in resource-limited settings.

## Introduction

Cranioplasty (CP) is a neurosurgical procedure primarily performed to reconstruct skull defects following decompressive craniectomy (DC), a lifesaving intervention typically used for conditions such as traumatic brain injury (TBI), malignant cerebral infarction, or hemorrhagic stroke. Beyond restoring cranial aesthetics and structural integrity, CP plays a pivotal role in improving cerebral hemodynamics, CSF dynamics, and neurological function [1,2].

The optimal timing of CP remains debated, with mixed results from prior studies (early < 90 days; late ≥ 90 days). Several studies suggest that early CP may facilitate better neurological recovery and reduce complications such as hydrocephalus and syndrome of the trephined [3,4]. However, others have raised concerns about increased infection risk and surgical complexity with earlier intervention [5,6]. Recent reviews suggest that timing alone is insufficient to predict outcomes, emphasizing the importance of patient-specific factors [7].

One such patient-specific variable is the collapse ratio, a radiographic measure of the degree of brain parenchymal depression at the craniectomy site. The collapse ratio quantitatively represents brain surface deviation relative to the skull contour, with positive values indicating parenchymal collapse and negative values reflecting brain bulging or residual swelling. Recent studies have shown that a higher collapse ratio is independently associated with worse postoperative outcomes, including increased rates of infection, seizures, hydrocephalus, and the need for revision surgery [8-10]. Huo et al. and Chen et al. emphasized that the collapse ratio may be a more reliable predictor of complications than surgical timing alone [8,9]. Incorporating this parameter into preoperative planning may therefore enhance clinical outcomes.

Additionally, other factors such as operative duration, the presence of skull concave fractures, and the underlying etiology (e.g., TBI) have been associated with complication rates following CP [11,12]. For instance, prolonged operative time has been linked to higher infection risk and poorer outcomes, potentially due to increased intraoperative blood loss and tissue exposure [13]. Similarly, TBI-related CP cases often present with more severe injuries and a higher incidence of subdural hematomas, which have been associated with an elevated risk of postoperative epilepsy [14,15].

Technological advancements have introduced promising solutions, such as 3D-printed scaffolds and bioactive materials, aimed at improving bone regeneration and reducing infection rates [16-18]. While these innovations continue to evolve, their integration into routine practice remains limited in resource-constrained settings, underscoring the need for reliable clinical and radiological predictors, such as the collapse ratio, to optimize patient selection and surgical planning.

The objective of this study was to determine whether preoperative collapse ratio and operative duration independently predict postoperative complications following CP, compared with traditional timing-based criteria. This retrospective study analyzed radiological, surgical, and clinical variables to identify independent predictors of adverse outcomes and to improve decision-making strategies for safer and more effective CP.

## Materials and methods

This retrospective observational single-center cohort study was conducted at Neuro Cardio and Multispeciality Hospital, a tertiary neurosurgical center in Biratnagar, Nepal.

Patient

A total of 100 patients who underwent CP following DC between 2018 and 2024 were included in this study. Patients were identified retrospectively through institutional electronic medical records, operative logbooks, and radiological imaging systems. Inclusion criteria were age ≥16 years, a unilateral skull defect resulting from DC, first-time CP, and sufficient preoperative imaging to allow measurement of the collapse ratio. Exclusion criteria were loss to follow-up, absence of a CT scan within two weeks prior to or 24 hours after CP, prior CP, or presence of an intracranial tumor. Cases with incomplete demographic, operative, or radiological data were also excluded to maintain data integrity. Patients were categorized into complication and non-complication groups based on a six-month follow-up.

Clinical management

The timing of CP was determined by the attending neurosurgical team based on individual patient factors, including neurological recovery, scalp wound healing, radiological resolution of cerebral edema, and infection status. Early procedures were performed selectively to address the syndrome of the trephined, facilitate cosmetic reconstruction, or promote neurological recovery, depending on the patient’s condition and the surgeon’s judgment.

A standardized clinical protocol was followed for all patients. All cranioplasties in this series used polymethyl methacrylate (PMMA) as the implant material. The PMMA was hand-molded intraoperatively, allowed to polymerize, and then fixed with titanium plates and screws. Standard neurosurgical sterile protocols were observed, and perioperative antibiotic prophylaxis was administered.

CP was performed via the original incision under general anesthesia. A subdural drainage tube was inserted during surgery and removed 48 hours postoperatively. Postoperative complications were assessed using a brain CT scan within 24 hours of the procedure. Routine postoperative care included monitoring for early complications such as surgical site infection, hematoma, seizures, hydrocephalus, and the need for revision surgery.

Data collection

Demographic information, including age and sex, was recorded, along with the primary diagnosis leading to DC, side of the skull defect, collapse ratio, DC-CP interval, operative duration, and postoperative complications (surgical site infection, epidural hemorrhage, subdural hemorrhage, hydrocephalus, and epilepsy).

The timing of CP was calculated from the date of DC to the date of CP, recorded in days, and categorized as early (<90 days) or late (≥90 days), consistent with prior literature and institutional practice [3,7]. Pre-CP non-contrast CT scans were evaluated to determine the collapse ratio using 3D-Slicer software (version 5.8.1, National Institutes of Health) for image reconstruction and measurement.

**Figure 1:**
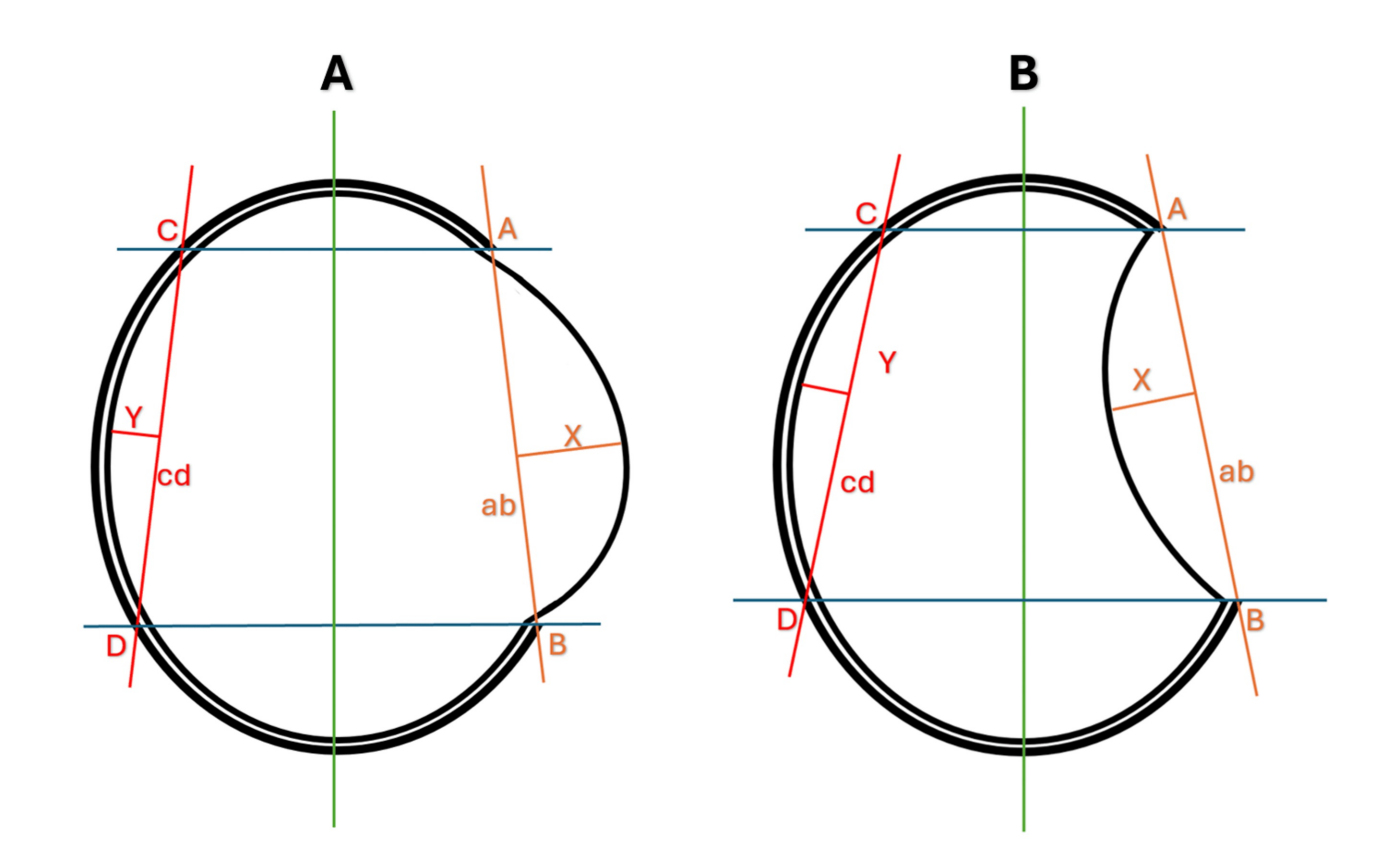
Illustrative measurement of the collapse ratio on preoperative CT images (A) Expansion of brain tissue. (B) Collapse of brain tissue. On the CT slice showing the largest bone window defect, the midline (green) was adjusted, and points A and B were marked at the edges of the defect. Perpendicular blue lines were drawn from points A and B to intersect the skull at points C and D. The connecting lines “ab” (orange) and “cd” (red) represent the cranial contour at the defect and on the contralateral side, respectively. X indicates the lowest (or highest) point of cortical indentation within the defect, and Y indicates the corresponding point on the contralateral intact brain. The outer black line represents the bone window, and the inner black line delineates the brain cortex. The collapse ratio was calculated as ((Y - X)/Y), reflecting the magnitude and direction of cortical deformation before CP. Positive values indicate brain sinking or depression beneath the defect, while negative or near-zero values indicate outward bulging or a flaccid brain. CP, cranioplasty Image credit: Rudra N. Shah

The CT slice showing the largest bone window defect was selected, and the midline (green line) was adjusted. Points A and B were marked at the edges of the cranial defect. From points A and B, perpendicular blue lines were drawn to intersect the intact skull on the contralateral side at points C and D. The line connecting A and B was labelled “ab” (orange line), and the line connecting C and D was labelled “cd” (red line). A perpendicular line from the lowest (in cases of brain collapse) or highest (in cases of brain expansion) point of the cortical surface within the defect to line AB was measured as X. The corresponding perpendicular distance from the contralateral intact cortical surface to line CD was measured as Y. The collapse ratio was calculated as (Y -X)/Y (Figure 1A, 1B). This ratio reflects both the magnitude and direction of cortical deformation: positive values indicate brain sinking beneath the defect, while negative or near-zero values indicate outward bulging or a flaccid brain.

Two independent reviewers measured the collapse ratio to ensure reliability. Inter-rater reliability was evaluated in 20 randomly selected cases using a two-way random-effects intraclass correlation coefficient (ICC(2,1)), demonstrating excellent agreement (ICC = 0.93).

Postoperative complications, including epidural or subdural hematoma, surgical site infection, hydrocephalus requiring shunt, epilepsy, and revision surgery, were recorded based on clinical evaluation, laboratory findings, and CT imaging. Follow-up varied, but all patients underwent at least one clinical and radiological assessment within three months post-CP and were followed for up to six months. No patients were lost to follow-up during this period.

Statistical analysis

Data were entered and cleaned using Microsoft Excel (2024; Microsoft Corporation, Redmond, WA, USA), and statistical analyses were performed using IBM SPSS Statistics for Windows, Version 30.0 (Released 2024; IBM Corp., Armonk, NY, USA). Continuous variables were summarized as mean ± SD and compared using independent t-tests. Categorical variables were expressed as frequencies and percentages, with comparisons made using the chi-square (χ²) or Fisher’s exact test, as appropriate.

Binary logistic regression was used to identify independent predictors of postoperative complications, reporting ORs with 95% CIs for collapse ratio, CP operative duration, and DC-CP interval. Continuous variables (collapse ratio, operative duration, and DC-CP interval) were entered into the regression model as continuous predictors, while the dependent variable was binary (presence or absence of postoperative complication). A stepwise binary logistic regression approach was applied, including variables with p < 0.10 on univariate testing.

Receiver operating characteristic (ROC) curve analysis, including calculation of the area under the curve (AUC), was conducted to evaluate the predictive value of collapse ratio and CP operative duration for postoperative complications. Variable selection for the regression model was determined a priori, based on clinical relevance and prior studies reporting determinants of post-CP complications [8,9]. Variables with p < 0.10 in univariate analyses were included in the multivariate logistic regression. Multicollinearity was assessed using variance inflation factors (VIF), all of which were <2, confirming the stability of the regression model. A p-value < 0.05 was considered statistically significant.

Ethical approval

The study was approved by the Nepal Health Research Council (NHRC) through its expedited review process, with ethical clearance granted under approval letter (reg. no. 373_2025). Informed consent was waived due to the retrospective nature of the study and the use of anonymized patient data. The study adhered to the ethical standards of the NHRC, international research guidelines, and the principles of the Declaration of Helsinki.

## Results

A total of 100 patients who underwent CP following DC were included in this study. Patients were divided into two groups: complication and non-complication. The mean age of patients who developed complications was 28.57 ± 12.26 years, compared to 33.19 ± 12.02 years in those without complications; this difference was not statistically significant (p = 0.329). All patients in the complication group were male (7/7, 100%), compared to 69/93 (74.2%) in the non-complication group; this difference was also not statistically significant (p = 0.136) (Table 1).

**Table 1:** Distributions of study patients by demographic variables (n = 100) ^a^ p-Value calculated using an unpaired t-test ^b^ p-Value calculated using Fisher’s exact test ns, not significant

Demographics	Complication (n = 7)		No complication (n = 93)		Test statistics	p-Value
	n	%	n	%		
Age in years						
<20	3	42.9	14	15.2	t(98) = 0.99	^a^0.329^ns^
21-30	1	14.3	30	32.4		
31-40	2	28.6	24	26.1		
≥60	1	14.3	25	27.3		
Mean ± SD	28.57 ± 12.26		33.19 ± 12.02			
Ranged (min-max)	17-50		3.5-60			
Gender						
Male	7	100	69	74.2	Fisher’s exact	^b^0.136^ns^
Female	0	0	24	25.8		

Among the different indications for craniectomy, TBI was present in all complication cases (7/7, 100%), whereas 84/93 (90.3%) of the non-complication group had TBI. Other causes, including hypertensive cerebral hemorrhage, aneurysmal hemorrhage, or infarction, were observed only in the non-complication group; however, differences in indications were not statistically significant (p = 0.505). Regarding the side of CP, 4/7 (57.1%) of patients with complications had right-sided defects compared to 41/93 (44.1%) in the non-complication group; this difference was not significantly associated with complications (p = 0.228) (Table 2).

**Table 2:** Distribution of study patients by craniectomy indication (n = 100) p-Value calculated using Fisher’s exact test ns, not significant; TBI, traumatic brain injury

Craniectomy indication	Complication (n = 7)		No complication (n = 93)		p-Value
	n	%	n	%	
TBI					
Yes	7	100	84	90.3	0.505^ns^
No	0	0	9	9.7	
Hypertensive cerebral hemorrhage					
Yes	0	0	3	3.2	0.802^ns^
No	7	100	90	96.8	
Aneurysmal cerebral hemorrhage					
Yes	0	0	4	4.3	0.744^ns^
No	7	100	89	95.7	
Arteriovenous malformation					
Yes	0	0	0	0	-
No	7	100	93	100	
Cerebral infarction					
Yes	0	0	2	0	1.0^ns^
No	7	100	93	100	
Skull defect side					
Left	3	42.9	56	60.2	0.228^ns^
Right	4	57.1	41	44.1	

Among patients with TBI, traumatic subdural hematoma was more common in the complication group (6/7, 85.7%) than in the non-complication group (52/84, 61.9%), although this difference was not statistically significant. In contrast, skull concave fractures were observed significantly more frequently in the complication group (4/7, 57.1%) compared to the non-complication group (18/84, 21.4%), with a p-value of 0.0487 (Table 3).

**Table 3:** Distribution of study patients by pathological types among TBI cases (n = 91) p-Value calculated using Fisher’s exact test ns, not significant; s, significant; TBI, traumatic brain injury

Pathological types with TBI	Complication (n = 7)		No complication (n = 84)		p-Value
	n	%	n	%	
Traumatic subdural hematoma					
Yes	6	85.7	52	61.9	0.4109^ns^
No	1	14.3	32	38.1	
Traumatic epidural hematoma					
Yes	1	14.3	14	16.7	1.000^ns^
No	6	85.7	69	83.3	
Traumatic intracranial hematoma					
Yes	0	0	13	15.5	0.5897^ns^
No	7	100	71	84.5	
Skull concave fracture					
Yes	4	57.1	18	21.4	0.0487^s^
No	3	42.9	66	78.6	

Epilepsy was the only postoperative complication recorded and was present in all complication cases (7/7, 100%) but in none of the non-complication group. No cases of infection, hydrocephalus, or bone resorption were observed during the six-month follow-up. None of the patients who developed postoperative epilepsy had documented seizure episodes prior to CP. Among the complication group, two patients underwent redo surgery. The difference in epilepsy incidence between the two groups was statistically significant (p < 0.05) (Table 4).

**Table 4:** Distribution of study patients by postoperative complications (n = 100) p-Value calculated using Fisher’s exact test s, significant

Types of postoperative complications	Complication (n = 7)		No complication (n = 93)		p-Value
	n	%	n	%	
Epilepsy					
Yes	7	100	0	0	0.001^s^
No	0	0	93	100	
Subdural effusion					
Yes	0	0	0	0	-
No	7	100	93	100	
Epidural hemorrhage					
Yes	0	0	0	0	-
No	7	100	93	100	
Hydrocephalus					
Yes	0	0	0	0	-
No	7	100	93	100	
Skin infection at the surgical site					
Yes	0	0	0	0	-
No	7	100	93	100	

The mean collapse ratio was significantly higher in the complication group (0.78 ± 0.16) compared to the non-complication group (0.36 ± 0.14; p < 0.001). The interval between DC and CP was shorter among patients who developed complications (2.43 ± 1.72 months) than in those who did not (6.52 ± 7.37 months), but this difference was not statistically significant (p = 0.148). The duration of the CP procedure was significantly longer in the complication group, averaging 139.71 ± 19.25 minutes compared to 115.08 ± 14.48 minutes in the non-complication group (p < 0.001) (Table 5).

**Table 5:** Predictors of postoperative complications after CP (n = 100) with logistic regression analysis CP, cranioplasty; ns, not significant; s, significant

Predictor	Complication (n = 7)	No complication (n = 93)	OR, 95% CI	p-Value
	Mean ± SD	Mean ± SD		
Collapse ratio	0.78 ± 0.16	0.36 ± 0.14	1.87(0.12-21.15)	0.001^s^
Ranged (min-max)	0.33-0.92	0.13-0.56		
CP operation duration (min)	2.43 ± 1.72	6.52 ± 7.37	1.68 (0.25-11.02)	0.001^s^
Ranged (min-max)	1-6	1-44		
DC-CP interval (months)	139.71 ± 19.25	115.08 ± 14.48	1.43 (0.52-3.97)	0.486^ns^
Ranged (min-max)	109-158	91-140		

Logistic regression analysis demonstrated that a higher collapse ratio (OR 1.87, p = 0.001) and longer CP operative duration (OR 1.68, p = 0.001) were independently associated with increased odds of postoperative complications. The DC-CP interval was not a statistically significant predictor in the adjusted model (Table 5). No evidence of multicollinearity was detected among predictor variables (all VIF < 2), supporting the stability of the multivariate model.

**Figure 2:**
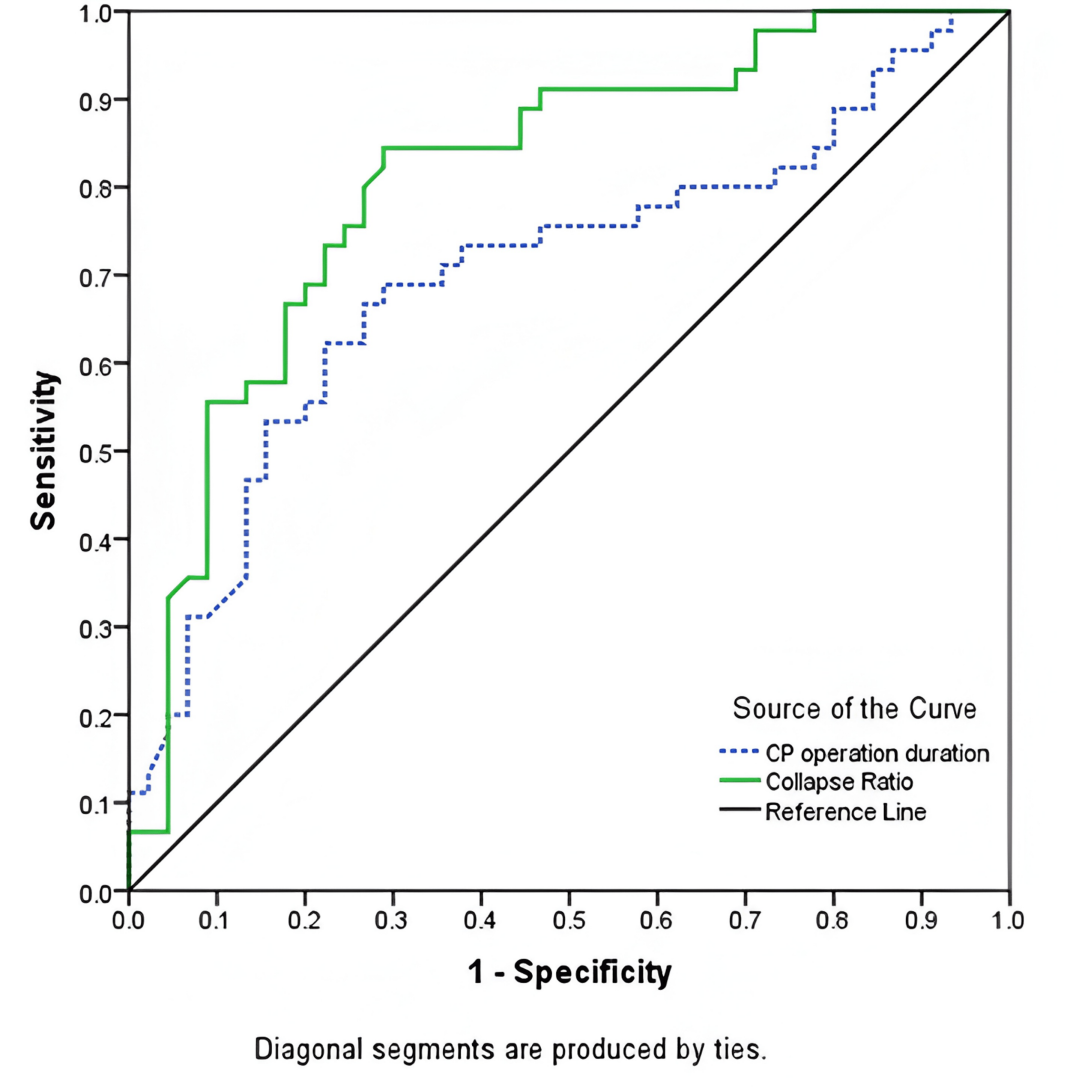
ROC curves comparing the predictive capability of collapse ratio (solid green line) and CP operative duration (dashed blue line) for post-CP complications CP, cranioplasty; ROC, receiver operating characteristic

ROC curve analysis demonstrated that the collapse ratio had excellent predictive capability for postoperative complications, with an AUC of 0.812 (p = 0.001), 88.4% sensitivity, and 55.6% specificity at a cutoff value of 0.39. CP operative duration showed moderate predictive ability, with an AUC of 0.702 (p = 0.004), a cutoff of 125 minutes, 73.3% sensitivity, and 60% specificity (Table 6, Figure 2).

**Table 6:** ROC analysis of collapse ratio and CP operative duration for predicting postoperative complications (n = 100) CP, cranioplasty; ROC, receiver operating characteristic

Predictors	Cutoff value	Sensitivity	Specificity	Area under the ROC curve	p-Value	95% CI	
						Lower bound	Upper bound
Collapse ratio	0.39	84.4	55.6	0.812	0.001^s^	0.722	0.902
CP operation duration (minutes)	125	73.3	60	0.702	0.004^s^	0.592	0.812

## Discussion

This retrospective study identified two independent predictors of early postoperative complications after CP following DC: a higher preoperative collapse ratio and prolonged operative duration. In contrast, the interval between DC and CP, although shorter among patients with complications, was not statistically significant in multivariate analysis. These findings suggest that patient-specific radiological and operative parameters may be more relevant than fixed timing thresholds when planning CP.

The collapse ratio, an objective imaging measure of the degree of brain parenchymal depression relative to the skull defect, has emerged as a valuable preoperative marker. In this study, a positive collapse ratio indicated cortical sinking (brain collapse), whereas negative values would reflect residual swelling. All patients in our cohort demonstrated positive values, reflecting varying degrees of brain collapse prior to CP. The collapse ratio likely reflects a combination of cortical atrophy, altered CSF hydrodynamics, venous outflow changes, and scar formation [8,9,19]. In our cohort, the mean collapse ratio in the complication group was more than double that of the non-complication group (0.78 vs. 0.36), showing strong predictive performance (AUC = 0.812) with an optimal threshold of 0.39. These findings align with previous studies, including Huo et al., who identified a collapse ratio ≥0.65 as a predictor of adverse postoperative outcomes, and Chen et al., who reported that CP timing was not an independent predictor of events such as seizures, hydrocephalus, or infection [8,9,19]. Unlike these large multicenter studies, our cohort represents a South Asian single-center population with uniform perioperative protocols, emphasizing both the broader generalizability of the collapse ratio and population-specific nuances.

Importantly, a preoperative collapse ratio exceeding 0.6 was associated with a markedly increased risk of complications. In such patients, delaying CP or optimizing CSF dynamics prior to surgery may reduce intraoperative tension and the likelihood of postoperative seizures, highlighting a potential practical application of the collapse ratio in routine surgical planning.

Our results also support growing evidence that rigid early-versus-late timing thresholds have limited predictive value compared with individualized patient parameters [3-7,20-22]. Although complications tended to occur in early CP, timing was not independently associated with adverse outcomes after adjusting for collapse ratio and operative factors.

Operative duration emerged as the second independent predictor of complications. Longer surgeries can increase contamination risk, anesthetic exposure, and tissue trauma and often reflect intraoperative complexity arising from concave fractures, dural adhesions, or distorted anatomy [13,14,23]. In our study, concave fractures were significantly more frequent in the complication group, echoing findings by Nguyen et al. and Hawryluk et al., who reported worse reconstruction outcomes in such cases [14,23]. These observations suggest that anticipated complexity should prompt meticulous preoperative planning, including the use of pre-shaped implants, templating, and an experienced surgical team to minimize operative time.

Epilepsy was the only early postoperative complication recorded, affecting all patients in the complication group. The exclusive manifestation of epilepsy may reflect the limited six-month follow-up, which favors the detection of early complications but may miss delayed events such as infection, bone resorption, or hydrocephalus. This is consistent with the recognized association between TBI, cortical injury, and postoperative seizures [14,24]. Sudden changes in intracranial pressure and cerebral perfusion following CP may precipitate seizures in susceptible brains [25]. These findings support closer intraoperative and postoperative neurological monitoring and, in selected high-risk patients, consideration of short-term antiepileptic prophylaxis [25].

Interestingly, no cases of surgical site infection were identified in the early postoperative period, despite infection being a commonly reported CP complication [5,13,26]. This may reflect strict adherence to sterile protocols, the use of PMMA implants, and consistent perioperative antibiotic prophylaxis. However, the relatively short follow-up limits the detection of delayed infections or bone flap resorption [26,27]. Redo CP was required in 2/7 (28.6%) patients with complications, comparable to rates reported by Yao et al. [26].

Overall, these findings support a shift from rigid timing-based protocols toward a tailored strategy incorporating radiological markers such as collapse ratio, anticipated operative complexity, and underlying pathology. In resource-limited settings, this approach could optimize outcomes without relying on high-cost technologies.

This study has several limitations. Its retrospective, single-center design introduces potential selection and classification bias, which may have led to under-reporting of minor or delayed complications. The small number of complications (7/100, 7%) limits the statistical power of regression and ROC analyses, increasing the risk of type II error. All complications occurred in male patients, introducing potential sex-related bias and limiting generalizability. Follow-up was limited to six months, precluding assessment of late outcomes such as bone flap resorption or long-term seizure control. Implant material was not randomized, and all procedures followed a uniform institutional protocol, which may affect generalizability. Prospective, multicenter studies with long-term follow-up are warranted to validate collapse ratio thresholds, assess their interaction with timing and implant type, and evaluate targeted perioperative interventions in high-risk patients.

## Conclusions

In this retrospective cohort study of patients undergoing CP after DC, a higher preoperative collapse ratio and prolonged operative duration were independently associated with early postoperative complications, particularly seizures. The interval between craniectomy and CP was not an independent predictor once these factors were accounted for. These findings underscore the value of incorporating quantitative imaging metrics, such as the collapse ratio, alongside operative planning considerations to better stratify risk and guide surgical decision-making. By identifying high-risk patients preoperatively, surgeons can implement strategies to streamline operative workflow, optimize neurological protection, and potentially improve outcomes. Prospective multicenter studies are warranted to validate these predictors, refine threshold values, and evaluate targeted interventions for complication prevention.
